# A validation study of the kidney failure risk equation in advanced chronic kidney disease according to disease aetiology with evaluation of discrimination, calibration and clinical utility

**DOI:** 10.1186/s12882-021-02402-1

**Published:** 2021-05-24

**Authors:** Ibrahim Ali, Rosemary L. Donne, Philip A. Kalra

**Affiliations:** 1 Department of renal medicine, Salford Royal NHS Foundation Trust, Stott Lane, Salford, M6 8HD UK; 2 Division of Cardiovascular Sciences, University of Manchester, Manchester, M13 9PL UK

**Keywords:** Kidney failure risk equation, Risk prediction, Chronic kidney disease, Discrimination, Calibration, Decision curve analysis, End-stage renal disease

## Abstract

**Background:**

The Kidney Failure Risk Equation (KFRE) predicts the 2- and 5-year risk of end-stage renal disease (ESRD) in patients with chronic kidney disease (CKD) stages 3a-5. Its predictive performance in advanced CKD and in specific disease aetiologies requires further exploration. This study validates the 4- and 8-variable KFREs in an advanced CKD population in the United Kingdom by evaluating discrimination, calibration and clinical utility.

**Methods:**

Patients enrolled in the Salford Kidney Study who were referred to the Advanced Kidney Care Service (AKCS) clinic at Salford Royal NHS Foundation Trust between 2011 and 2018 were included. The 4- and 8-variable KFREs were calculated on the first AKCS visit and the observed events of ESRD (dialysis or pre-emptive transplantation) within 2- and 5-years were the primary outcome. The area under the receiver operator characteristic curve (AUC) and calibration plots were used to evaluate discrimination and calibration respectively in the whole cohort and in specific disease aetiologies: diabetic nephropathy, hypertensive nephropathy, glomerulonephritis, autosomal dominant polycystic kidney disease (ADPKD) and other diseases. Clinical utility was assessed with decision curve analyses, comparing the net benefit of using the KFREs against estimated glomerular filtration rate (eGFR) cut-offs of < 20 ml/min/1.73m^2^ and < 15 ml/min/1.73m^2^ to guide further treatment.

**Results:**

A total of 743 patients comprised the 2-year analysis and 613 patients were in the 5-year analysis. Discrimination was good in the whole cohort: the 4-variable KFRE had an AUC of 0.796 (95% confidence interval [CI] 0.762–0.831) for predicting ESRD at 2-years and 0.773 (95% CI 0.736–0.810) at 5-years, and there was good-to-excellent discrimination across disease aetiologies. Calibration plots revealed underestimation of risk at 2-years and overestimation of risk at 5-years, especially in high-risk patients. There was, however, underestimation of risk in patients with ADPKD for all KFRE calculations. The predictive accuracy was similar between the 4- and 8-variable KFREs. Finally, compared to eGFR-based thresholds, the KFRE was the optimal tool to guide further care based on decision curve analyses.

**Conclusions:**

The 4- and 8-variable KFREs demonstrate adequate discrimination and calibration for predicting ESRD in an advanced CKD population and, importantly, can provide better clinical utility than using an eGFR-based strategy to inform decision-making.

**Supplementary Information:**

The online version contains supplementary material available at 10.1186/s12882-021-02402-1.

## Introduction

Chronic kidney disease (CKD) is not a benign condition given worsening kidney function is an independent risk factor for progression to end-stage renal disease (ESRD), cardiovascular events and all-cause mortality [1]. Accurately predicting ESRD is a cornerstone of optimal CKD care as it enables targeted treatment in high-risk patients, including supporting better risk communication with patients and appropriate prioritisation of treatment pathways that include education regarding renal replacement therapies (RRT), especially the benefits of pre-emptive living donor kidney transplantation [2–4].

To date, the Kidney Failure Risk Equation (KFRE) remains the most well-validated risk prediction tool, predicting the 2- and 5-year risk of progression to ESRD in patients with CKD stages 3a-5 [5]. The 4-variable KFRE requires age, sex, estimated glomerular filtration rate (eGFR) and albuminuria, whilst the 8-variable KFRE incorporates the additional parameters of serum calcium, phosphate, albumin and bicarbonate. Not only has the KFRE been shown to be accurate for risk prediction but absolute risk thresholds have been implemented into clinical care systems, such as a 2-year ESRD risk of ≥40% to guide dialysis access planning in patients who have chosen future dialysis [6].

Whilst the KFRE appears a promising aid to decision-making, there is a lack of evidence regarding its ability to risk predict in more advanced CKD and in specific disease aetiologies, which are known to progress at different trajectories. Thus far, the only study to have explored this was by Hundemer et al. [7], who validated the 4-variable KFRE in a Canadian cohort of patients referred to a multi-disciplinary pre-dialysis clinic. They showed the KFRE adequately predicted ESRD in this cohort with a median eGFR of 15 ml/min/1.73m^2^ (interquartile range: 12-19 ml/min/1.73m^2^), irrespective of whether patients had diabetic nephropathy, hypertensive nephropathy, glomerulonephritis, autosomal dominant polycystic kidney disease (ADPKD) or other conditions. However, the authors did not validate the predictive performance of the 8-variable KFRE, which may be of particular relevance in advanced CKD given the potential prognostic importance for mineral-bone disease, acidosis and inflammation at CKD stages 4–5, and which are captured by the extra parameters of the 8-variable KFRE. Furthermore, whilst statistical measures of model performance were reported, such as discrimination and calibration, the clinical utility of the KFRE was not evaluated. However, measures of utility are recognised as a useful marker of prediction model performance [8].

In light of the work by Hundemar et al. [7], and to address gaps in the literature, we undertook a validation study of the KFRE in order to 1) provide insight, to the best of our knowledge for the first time, on the predictive accuracy of both the 4- and 8-variable KFRE in an advanced CKD cohort, stratified to disease aetiology, in the United Kingdom (UK); and 2) determine whether the KFREs could offer clinical utility, and thus provide evidence to develop a risk-based strategy to deliver care as opposed to one that relies on eGFR thresholds.

## Methods

### Study population and setting

A retrospective analysis was undertaken in patients in the Salford Kidney Study (SKS). The SKS is an ongoing observational study, which since 2002 has focused on recruiting patients with non-dialysis CKD. Patients referred to the renal services at Salford Royal NHS Foundation Trust (SRFT), a tertiary renal centre in the UK, who are aged 18 years or older with an eGFR< 60 ml/min/1.73m^2^ are eligible for enrolment. This study focused on patients in the SKS who were referred to the advanced kidney care service (AKCS) clinic in SRFT, a multidisciplinary clinic comprising doctors, specialist nurses and dieticians that provides holistic care for patients with advanced CKD. Patients are typically referred to the AKCS clinic once they reach an eGFR of < 20 ml/min/1.73m^2^ or if they have an eGFR of 20-30 ml/min/1.73m^2^ but are deemed to be rapidly progressing by the referring clinician. Emphasis in the AKCS clinic is placed on treating complications of CKD (such as anaemia, fluid retention and mineral bone disease) and educating patients about potential future treatment options. Opportunities for pre-emptive transplant are optimised by early discussion about living kidney donation, assessment of suitability for transplant at the first clinic visit and prompt referral to a dedicated one-stop transplant work-up clinic. The frequency of clinic visits and monitoring is largely guided by changes in patients’ symptoms and eGFR values and is at the discretion of the clinician in clinic.

### Data variables

The 4-variable KFRE requires age, sex, eGFR and uACR, whilst the 8-variable KFRE comprises these four variables along with serum calcium, phosphate, albumin and bicarbonate [4]. The eGFR was determined using the Chronic Kidney Disease Epidemiology Collaboration (CKD-EPI) equation. The following unit conversions were made to align measurements in the SKS to the original KFRE study: calcium, measured in mmol/L, was converted to mg/dL by multiplying values by 4; phosphate, measured in mmol/L, was converted to mg/dL by multiplying by 3.1; albumin, measured in g/L, was converted to g/dL by dividing values by 10. The urine protein:creatinine ratio (uPCR) units of mg/mmol were converted to mg/g by multiplying values by 8.84. The uACR was then derived from the uPCR for all patients using a validated conversion formula that has been shown to provide good discrimination when used with the KFRE (Additional file 1) [9]. All variables used in this present analysis were taken on each patient’s first attendance in the AKCS clinic.

### Cohort assembly

**Fig. 1:**
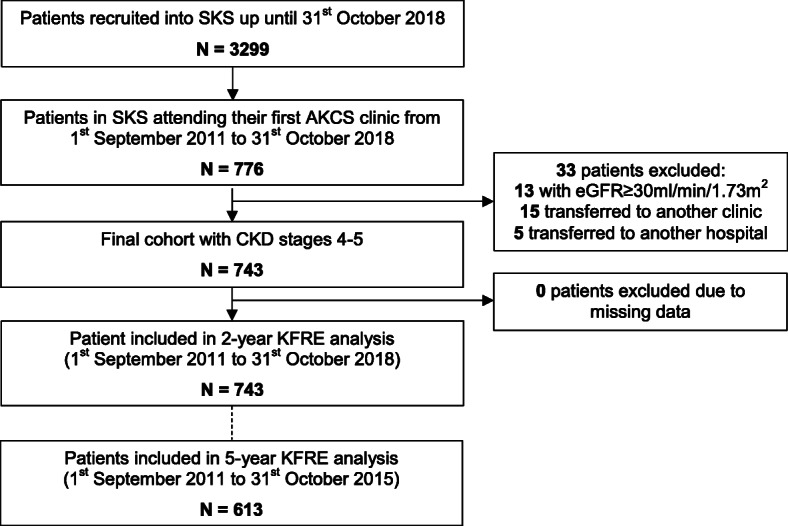
Study cohort assembly. Abbreviations: SKS (Salford Kidney Study); AKCS (Advanced Kidney Care Service); uPCR (urine protein:creatinine ratio); uACR (urine albumin:creatinine ratio); CKD (chronic kidney disease); eGFR (estimated glomerular filtration rate); KFRE (Kidney Failure Risk Equation)

Patients with an eGFR< 30 ml/min/1.73m^2^ who attended their first AKCS clinic from 1st September 2011 to 31st October 2018 were included in order to enable a minimum 2-year follow-up in all subjects, and this comprised the whole study cohort. To permit calculation of the 5-year risk of ESRD, only patients from within the whole cohort who had their first AKCS clinic visit from 1st September 2011 up until 31st October 2015 were included in the 5-year analysis. Patients were excluded if they were referred out of the AKCS clinic or transferred to other hospitals for ongoing care as the primary ESRD outcomes for these patients could not be determined. No patients were excluded due to missing data in our cohort (Fig. 1).

Patients were subdivided into five disease categories: diabetic nephropathy, hypertensive nephropathy, glomerulonephritis, autosomal dominant adult polycystic kidney disease (ADPKD), and all other causes. The diagnosis of diabetic or hypertensive nephropathy was based on either histological data or clinical judgement by the patient’s lead clinician. Patients were diagnosed with glomerulonephritis based on histology and those with ADPKD met international diagnostic guidelines.

### Study outcomes

The death-censored events of ESRD at 2- and 5-years, calculated using the calibrated non-North American 4- and 8-variable KFREs (Additional file 1), were the primary outcomes. ESRD was defined as initiation of haemodialysis, peritoneal dialysis, conservative care or receiving a pre-emptive renal transplant. A death-censored analysis was undertaken as this is in keeping with the original KFRE development study [4] but a sensitivity analysis that considered death prior to ESRD as a competing event was undertaken. Outcome data was evaluated until 1st November 2020.

### Statistical analysis

For baseline characteristics, continuous data is presented as median with interquartile ranges and categorical data as absolute numbers with percentages. The predictive performance of the 4- and 8-variable KFREs at 2- and 5-years were evaluated using discrimination and calibration metrics for the whole cohort and for patients in the five disease categories.

Discrimination, which is the extent a model can differentiate patients with or without the study outcome based on the risk score, was defined by the area under the curve (AUC) of a receiver operator characteristic curve (ROC), along with 95% confidence intervals [CI] [10]. Perfect discrimination amounts to an AUC of 1.0. We defined acceptable discrimination by an AUC of 0.6–0.7, good discrimination as an AUC of 0.7–0.8, whilst values > 0.8 represented excellent discrimination [11]. Pairwise comparisons of the AUCs were undertaken using DeLong’s method [12] to assess for differences in discrimination performance between the 4- and 8-variable KFREs in the whole cohort and between each disease group separately.

Calibration, the extent the predicted risk scores accurately estimate the observed values, was visually assessed by a calibration plot. Here, the predicted risk scores are plotted against the observed outcome of ESRD, which is treated as a binary outcome, and a smoothing function is then applied [13]. Perfect agreement between the predicted risks and observed events produces a calibration line of 45°.

Whilst discrimination and calibration provide statistical measures of performance, both fail to adequately describe the clinical utility of a model. To address this, a decision curve analysis can be undertaken that illustrates the impact of a risk model in supporting decision-making at various threshold probabilities [8, 14]. The threshold probabilities, plotted on the x-axis, represent the range of appropriate risk probabilities (identified beforehand) at which a model could guide treatment when compared to the default strategies of ‘treatment for all’ and ‘treatment for no-one’. For our study, the upper risk limit for the 2-year KFRE analysis was set at 40%, a criterion proposed as a suitable cut-off for deciding upon planning for dialysis access and transplantation [5, 6]. For the 5-year KFRE analysis, the upper limit was set at 50% [5]. A treatment can refer to a variety of measures including further investigations or initiation of a therapy. In our study, we denote treatment as increased frequency of monitoring and prioritisation of referral for kidney transplant or timely dialysis access planning. The net benefit, plotted on the y-axis of a decision curve analysis, takes account of the relationship between the number of true positive and false positive cases within the sample population across the pre-defined range of threshold probabilities and is given by the following equation:

$$ Net\  benefit=\left(\frac{True\ positive}{Total\ sample\ size}\right)-\left[\left(\frac{False\ positive}{Total\ sample\ size}\right)\times \left(\frac{Threshold\ probability}{1- threshold\ probability}\right)\right] $$

Net benefit, represented as true positive cases, can also be expressed as the number of unnecessary interventions avoided in a population by simply focusing on true negative cases. For our study, unnecessary interventions translate as identifying patients who would suit less intensive monitoring and for whom referral for transplantation or dialysis access planning could be delayed.

When comparing different prediction methods, the model with the highest net benefit on the y-axis across the range of threshold probabilities would be deemed to be of optimal value [14]. In this study, the utility of the 4- and 8-variable KFREs for risk prediction at 2- and 5-years was compared against an eGFR-based strategy to guide further treatment using cut-off values of an eGFR< 20 ml/min/1.73m^2^ and < 15 ml/min/1.73m^2^. In addition, the median time-to-ESRD was calculated for the optimal model to provide information on the appropriate timeframe for when dialysis access formation should be undertaken.

### Sensitivity analysis

Survival curves for the 4-variable KFREs were produced to compare the differences in outcome between a death-censored analysis and an analysis in which death prior to ESRD is handled as a competing event.

All statistical analyses were conducted using R, version 4.0.2 (The R Foundation for Statistical Computing Platform). A *p*-value of < 0.05 was considered statistically significant.

### Ethical approval

The SKS received ethical approval from the North West Greater Manchester South Research Ethics Committee (REC15/NW/0818). Written informed consent was obtained from all patients. The methods described herein were carried out in accordance with relevant guidelines and regulations of the SKS.

The reporting of this validation study complies with the TRIPOD (Transparent Reporting of a Multivariable Prediction Model for Individual Prognosis or Diagnosis) statement [15] (Additional file 2).

## Results

### Baseline characteristics

A total of 743 patients were included in the 2-year analysis of the 4- and 8-variable KFREs (Table 1). In this cohort, the median age was 68.5 years (56.9–77.1 years), and the majority of patients were male (62%) and was almost exclusively Caucasian (94%). The vast majority had a co-morbid diagnosis of hypertension (97%) and 40% of patients had diabetes. The most common disease-specific aetiology was diabetic nephropathy (24%). The median eGFR was 16 m/min/1.73m^2^ (13-18 ml/min/1.73m^2^),

**Table 1:** Baseline characteristics according to disease aetiology for all patients attending the AKCS clinic from 2011 to 2018

Variable	Whole cohort	Diabetic nephropathy	Hypertensive nephropathy	GN	ADPKD	Other diseases
Patient numbers	743	178	125	86	64	290
Age, years	68.5 (56.9–77.1)	66.1 (57.4–74.9)	76.3 (69.0–81.5)	62.6 (47.1–72.5)	54.3 (46.4–63.3)	71.2 (61.3–78.5)
Male, *n* (%)	462 (62)	118 (66)	83 (66)	55 (64)	38 (59)	165 (57)
Caucasian, *n* (%)	695 (94)	163 (92)	121 (97)	79 (92)	63 (98)	275 (95)
Hypertension, *n* (%)	723 (97)	176 (99)	125 (100)	85 (99)	61 (95)	276 (95)
Diabetes mellitus, *n* (%)	296 (40)	178 (100)	33 (26)	17 (20)	2 (3)	66 (23)
**Laboratory values**						
^a^eGFR, ml/min/1.73m^2^	16 (13–18)	16 (13–19)	15 (13–18)	16 (12–18)	16 (13–18)	15 (13–18)
^b^urine albumin:creatinine ratio, mg/g	409 (85–1356)	896 (245–2304)	172 (43–621)	1345 (496–2520)	130 (45–332)	362 (80–996)
^c^Calcium, mg/dL	9.32 (8.96–9.72)	9.36 (9.04–9.75)	9.20 (8.76–9.56)	9.28 (8.96–9.76)	9.32 (8.91–9.56)	9.40 (8.96–9.76)
^c^Phosphate, mg/dL	3.91 (3.41–4.53)	4.00 (3.44–4.62)	3.81 (3.32–4.50)	4.31 (3.60–4.86)	4.03 (3.57–4.35)	3.84 (3.32–4.37)
Bicarbonate, mEq/L	21.8 (19.6–24.4)	22.4 (20.3–25.5)	21.6 (19.7–23.8)	20.8 (19.2–23.5)	22.2 (19.1–23.8)	21.7 (19.4–24.4)
^d^Albumin, g/dL	4.2 (3.9–4.4)	4.0 (3.7–4.2)	4.2 (4.0–4.4)	4.0 (3.7–4.3)	4.4 (4.2–4.6)	4.2 (3.9–4.4)
**KFRE scores**						
4-variable 2-year score, %	24 (11–42)	31 (13–53)	15 (7–30)	39 (23–66)	19 (11–33)	22 (10–36)
4-variable 5-year score, %	65 (36–88)	76 (43–95)	47 (24–75)	85 (63–98)	56 (36–79)	61 (33–83)
8-variable 2-year score, %	20 (10–39)	23 (12–46)	15 (7–28)	31 (18–67)	18 (9–28)	19 (9–32)
8-variable 5-year score, %	64 (37–89)	68 (45–94)	53 (27–77)	81 (59–99)	60 (34–78)	61 (35–82)

Continuous data expressed as median (interquartile range) and categorical data as number (percentage)

*Abbreviations*: *AKCS* Advanced kidney care service clinic), *GN* glomerulonephritis, *ADPKD* autosomal dominant polycystic kidney disease, *eGFR* estimated glomerular filtration rate, *ESRD* end-stage renal disease, *KFRE* Kidney Failure Risk Equation

^a^eGFR was calculated using the Chronic Kidney Disease Epidemiology Collaboration (CKD-EPI) equation. ^b^urine albumin:creatinine ratios were acquired by converting urine protein:creatinine ratios using a validated formula [9]. ^c^Calcium and phosphate were measured in mmol/L and converted to mg/dL by multiplying values by 4 and 3.1 respectively. ^d^Albumin was measured in g/L and converted to g/dL by dividing by 10

which was similar across the disease categories, and the median uACR was 409 mg/g (85-1356 mg/g), which was comparatively higher in patients with glomerulonephritis and diabetic nephropathy than in patients with hypertensive nephropathy and ADPKD. All these characteristics were similar in the cohort of 613 patients, which comprised the 5-year analysis of the 4- and 8-variable KFREs (Additional file 3). A comparison of the baseline characteristics of the 2-year cohort with the original KFRE development cohort [4] is provided in Additional file 4.

### KFRE risk scores and outcome data

In the 2-year analysis, the median 2-year risk score for the 4- and 8-variable KFREs were similar at 24% (95% CI 11–42%) and 20% (95% CI 10–39%) respectively (Table 1), with the highest risk scores seen in patients with glomerulonephritis, followed by those with diabetic nephropathy. In the 5-year analysis cohort (Additional file 3), the median 4- and 8-variable 5-year risk scores were both 65% (95% CI of 36–88% for the 4-variable KFRE and 36–83% for the 8-variable KFRE). As per the 2-year analysis, the highest disease-specific 5-year risks for both the 4- and 8-variable KFREs were produced in those with glomerulonephritis followed by those with diabetic nephropathy.

Table 2 provides the outcome data for ESRD and death prior to ESRD in the whole cohort and across disease categories. For the 2-year analysis, 257 patients (35%) reached ESRD within 2-years, whilst 101 patients (14%) died prior to ESRD. In the 5-year analysis, 331 patients (54%) reached ERSD within 5-years and there were 164 deaths (27%) prior to ESRD.

**Table 2:** Outcome data for the analyses at 2-years and 5-years

	Whole cohort	Diabetic nephropathy	Hypertensive nephropathy	GN	ADPKD	Other diseases
**2-year outcomes**						
Patient numbers	743	178	125	86	64	290
ESRD, *n* (%)	257 (35)	65 (37)	32 (26)	40 (47)	40 (63)	80 (28)
Deaths prior to ESRD, *n* (%)	101 (14)	29 (16)	26 (21)	9 (10)	0 (0)	36 (12)
**5-year outcomes**						
Patient numbers	613	140	115	75	49	234
ESRD, *n* (%)	331 (54)	78 (56)	45 (39)	51 (68)	44 (90)	113 (48)
Deaths prior to ESRD, *n* (%)	164 (27)	47 (34)	42 (37)	13 (17)	3 (6)	59 (25)

*Abbreviations*: *GN* glomerulonephritis, *ADPKD* autosomal dominant polycystic kidney disease, *ESRD* end-stage renal disease

### KFRE discrimination performance

**Fig. 2:**
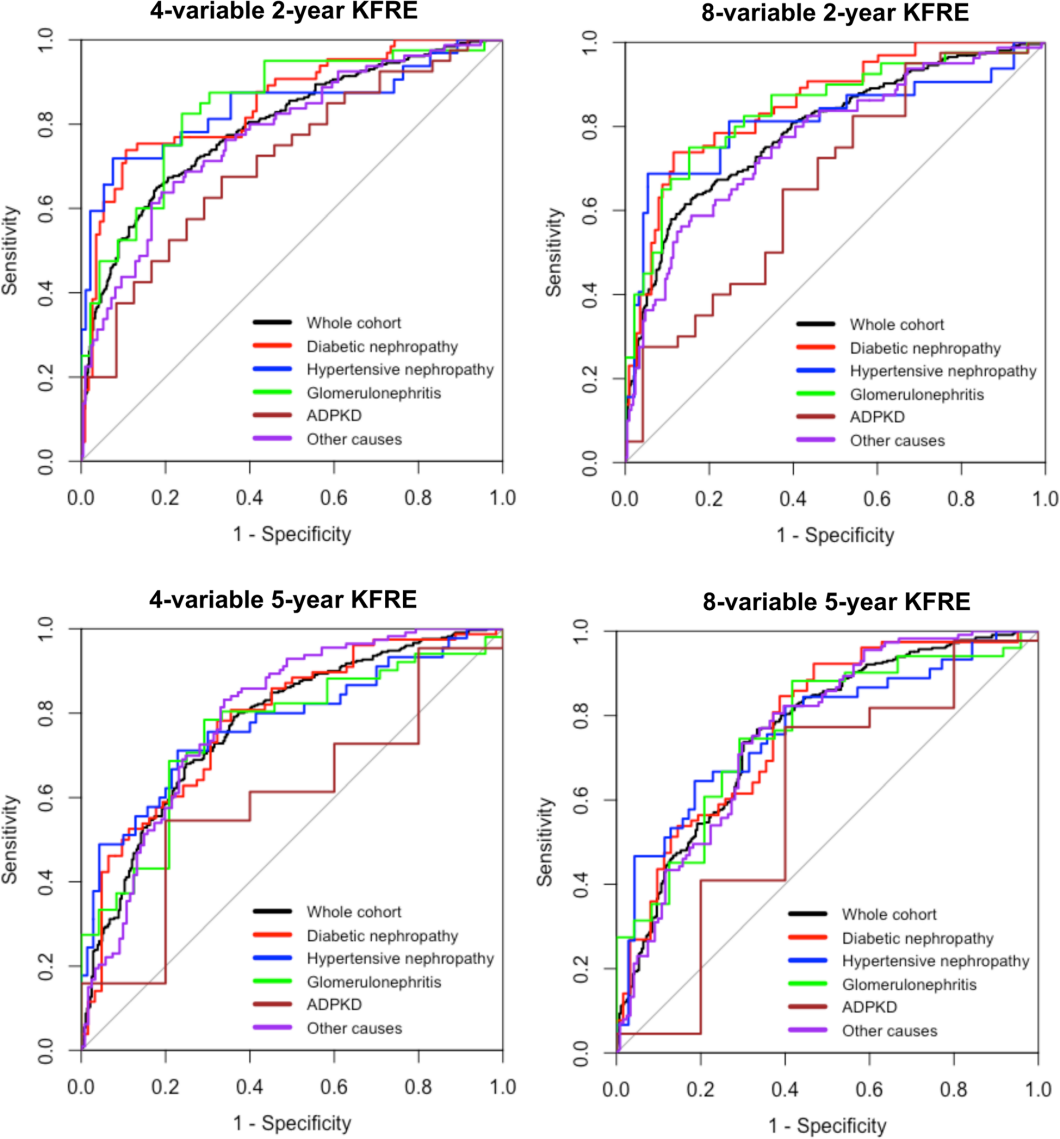
ROC curves for the 4- and 8-variable KFREs at 2- and 5-years according to disease aetiology. Abbreviations: ROC (receiver operator characteristic curve; KFRE (Kidney Failure Risk Equation)

Figure 2 shows the ROC curves for the 4- and 8-variable KFREs predicting risk at 2- and 5-years for the whole cohort and in each of the disease groups. A summary of the AUC values is provided in Table 3 for the 2-year analysis and in Table 4 for the 5-year analysis. In the 2-year analysis, the 4-variable KFRE had good discrimination in the whole cohort with an AUC of 0.796 (95% CI 0.762–0.831). It showed excellent discrimination for diabetic nephropathy at 0.850 (95% CI 0.789–0.910), hypertensive nephropathy at 0.841 (95% CI 0.744–0.938) and glomerulonephritis at 0.842 (95% CI 0.757–0.926), with good discrimination for ADPKD at 0.713 (95% CI 0.584–0.841) and for other diseases at 0.777 (95% CI 0.716–0.838). The 8-variable 2-year KFRE produced statistically similar AUC readings compared with the 4-variable 2-year values (Table 3).

**Table 3:** AUCs for the 2-year analysis of the 4- and 8-variable KFREs

Patients	4-variable 2-year risk AUC (95% CI)	8-variable 2-year risk AUC (95% CI)	***p***-value
Whole cohort	0.796 (0.762–0.831)	0.793 (0.758–0.828)	0.66
Diabetic nephropathy	0.850 (0.789–0.910)	0.856 (0.798–0.912)	0.72
Hypertensive nephropathy	0.841 (0.744–0.938)	0.814 (0.710–0.919)	0.07
Glomerulonephritis	0.842 (0.757–0.926)	0.843 (0.757–0.929)	0.96
ADPKD	0.713 (0.584–0.841)	0.668 (0.527–0.808)	0.18
Other diseases	0.777 (0.716–0.838)	0.770 (0.707–0.833)	0.73

Comparison between AUCs undertaken by DeLong’s method [12]

*Abbreviations*: *AUC* area under the receiver operator characteristic curve, *CI* confidence interval, *ADPKD* autosomal dominant polycystic kidney disease

**Table 4:** AUCs for the 5-year analysis of the 4- and 8-variable KFREs

Patients	4-variable 5-year risk AUC (95% CI)	8-variable 5-year risk AUC (95% CI)	***p***-value
Whole cohort	0.773 (0.736–0.810)	0.763 (0.725–0.800)	0.22
Diabetic nephropathy	0.783 (0.706–0.859)	0.776 (0.698–0.854)	0.71
Hypertensive nephropathy	0.774 (0.682–0.866)	0.769 (0.677–0.861)	0.76
Glomerulonephritis	0.755 (0.640–0.870)	0.764 (0.649–0.879)	0.70
ADPKD	0.600 (0.328–0.872)	0.605 (0.268–0.941)	0.95
Other diseases	0.790 (0.732–0.848)	0.763 (0.702–0.823)	0.09

Comparison between AUCs undertaken by Delong’s method [12]

*Abbreviations*: *AUC* area under the receiver operator characteristic curve, *CI* confidence interval, *ADPKD* autosomal dominant polycystic kidney disease

For the 5-year analysis, the 4-variable KFRE showed good discrimination in the whole cohort with an AUC of 0.773 (95% CI 0.736–0.810) and good discrimination was seen in the other disease categories except for ADPKD, which showed a much lower AUC of 0.600 (95% CI 0.328–0.872). These findings were similarly reproduced with the 8-variable 5-year calculations (Table 4).

Pairwise comparisons of all the ROC curves between each of the disease categories did not show any statistically significant differences except between patients with ADPKD compared with those with diabetic nephropathy and glomerulonephritis, but this only applied to the 8-variable 2-year KFRE (Additional file 5).

### KFRE calibration performance

**Fig. 3:**
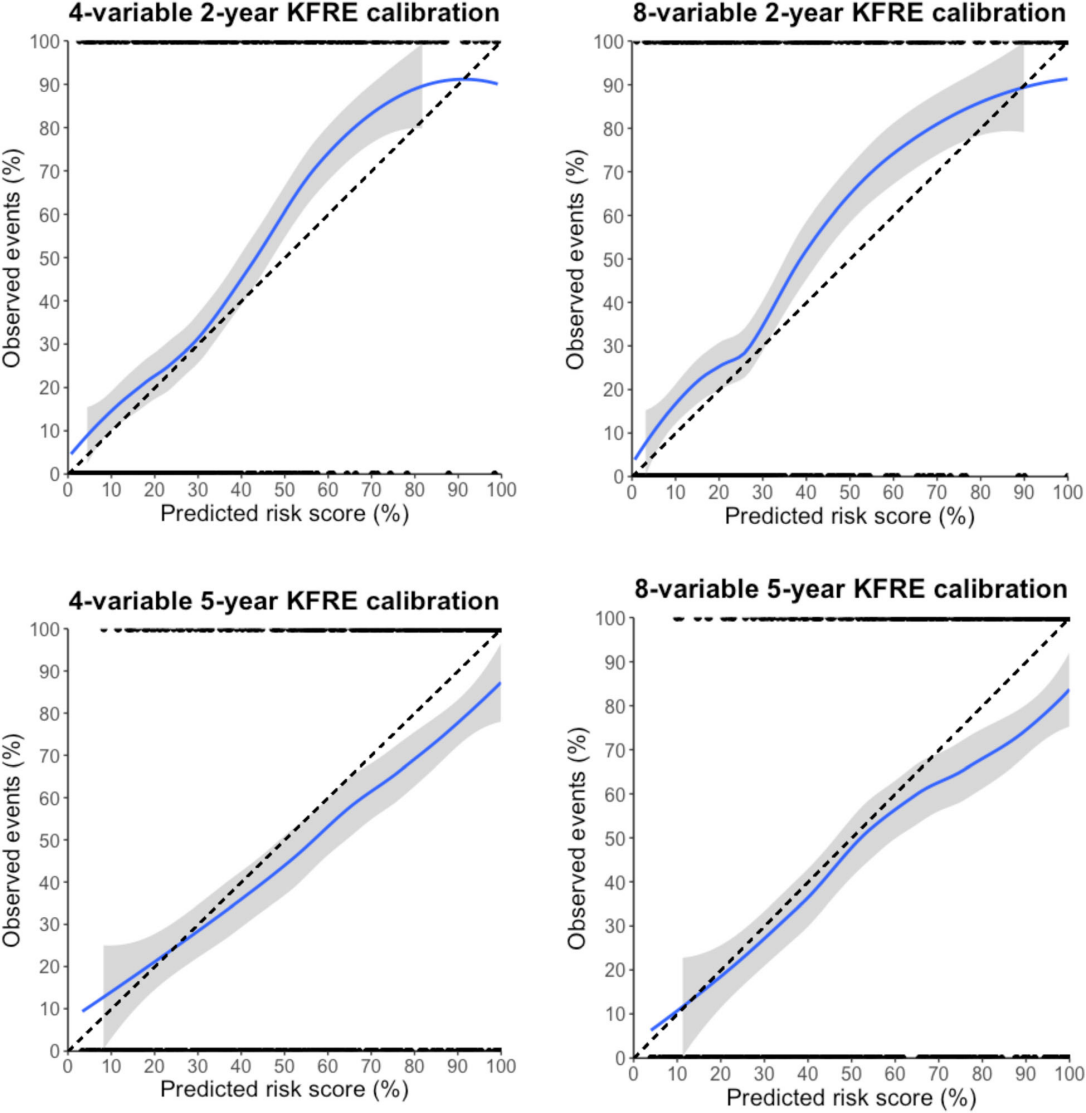
Calibration plots for the 4- and 8-variable KFREs at 2- and 5-years. A smoothing loess line has been applied to each graph. Grey shaded area represents 95% confidence intervals of the observed frequency of events. The black dots at 0% represent patients who did not develop ESRD and those at 100% represent patients who did develop ESRD

The calibration plots in Fig. 3 show adequate calibration for the 4- and 8-variable KFREs at 2- and 5-years but there was a tendency for underestimation of risk scores in the 2-year analysis, whereas overestimation of risk was more notably seen in the 5-year calibration plots for both the 4- and 8-variable KFREs. These differences in risk prediction were also borne out in the tabulated calibration data across disease aetiologies shown in Additional file 6, with the exception being patients with ADPKD, for whom the KFRE consistently underestimated the observed events in all calculations in the 2- and 5-year analyses.

### Clinical utility

**Fig. 4:**
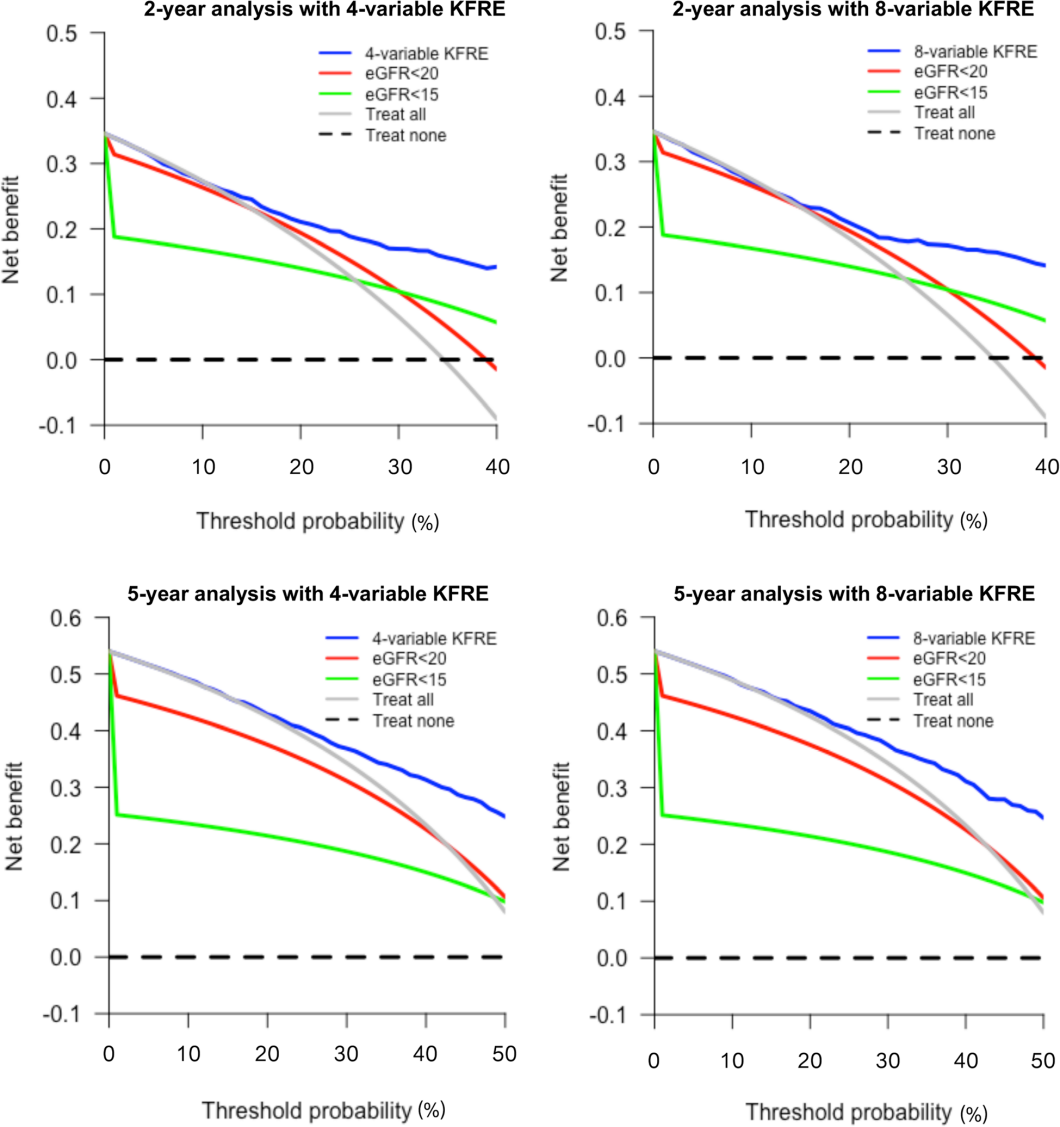
Decision curves analyses for the 4- and 8-variable KFREs at 2- and 5-years. The decision curves show that the both the 4- and 8-variable KFREs produced the highest net benefit for patients at 40% ESRD risk at 2-years and 50% ESRD risk at 5-years when compared to using eGFR thresholds of < 20 ml/min/1.73m^2^ and < 15 ml/min/1.73m^2^. Abbreviations: eGFR (estimated glomerular filtration rate), in ml/min/1.73m^2^; KFRE (Kidney Failure Risk Equation)

The decision analysis curves in Fig. 4 show the 4- and 8-variable KFREs are better for guiding further intervention at relevant threshold probabilities compared to using eGFR cut-offs at < 20 ml/min/1.73m^2^ and < 15 ml/min/1.73m^2^.

When compared with an eGFR cut-off of < 15 ml/min/1.73m^2^ at a 40% threshold probability, the 4-variable 2-year KFRE was able to identify an extra 8 patients per 100 that would progress to ESRD and identify 13 more patients per 100 for whom intervention could be delayed. The median time-to-ESRD for patients with a 2-year KFRE risk of ≥40% was approximately 11 months (6-19 months).

At a 50% risk threshold, the 4-variable 5-year KFRE identified 14 extra patients per 100 who would progress to ESRD and could identify delaying intervention in 14 more patients per 100 when compared with using an eGFR< 20 ml/min/1.73m^2^. In addition, it was able to identify 15 more true positive cases per 100 patients and 15 extra true negative cases compared with using an eGFR of < 15 ml/min/1.73m^2^ to guide further treatment. The median time-to-ESRD for patients with a 5-year KFRE risk of ≥50% was approximately 20 months (10-37 months).

The net benefit results of the 4-variable KFRE were similarly seen when using the 8-variable KFRE for 2- and 5-year risk prediction.

### Sensitivity analysis

The cumulative incidence of ESRD using the Kaplan-Meier survival curve, in which death prior to ESRD was censored, was compared to the cumulative incidence of ESRD when adjusted for death as a competing event. Using the 4-variable KFRE as the main example, Additional file 7 shows that the death-censored approach overestimates the probability of ESRD, which was especially apparent at 5-years follow-up.

## Discussion

This validation study shows that the use of the 4- and 8-variable KFREs can be of clinical utility in an advanced CKD population and offers evidence for switching towards a risk-based model of care above one that relies solely on eGFR thresholds to trigger intervention in high-risk patients.

We undertook a similar study to the one reported by Hundemer et al. [7], who recently provided a closer evaluation of the 4-variable KFRE in patients with advanced CKD and in specific disease categories, which had hitherto been lacking. The median eGFR of 15 ml/min/1.73m^2^ (12-19 ml/min/1.73m^2^) in their work closely matches the 16 ml/min/1.73m^2^ (13-18 ml/min/1.73m^2^) in ours and we share similar baseline patient characteristics of age and sex. The rates of ESRD were higher in their study compared to ours (42% and 64% reached ESRD at 2- and 5-years compared to 35% and 54% in our study), and this was reflected in higher KFRE risk scores. Nonetheless, we similarly found that patients with glomerulonephritis and diabetic nephropathy had the highest disease-specific risk scores whereas those with hypertensive nephropathy and ADPKD had the lowest. Our work extends upon the study by Hundemer et al. [7] with a geographical validation of the KFREs in a UK cohort and we provide the following four main contributions:

### The 4-variable KFRE is sufficient for risk prediction in advanced CKD

We show for the first time that the 8-variable KFRE performs on par with its 4-variable counterpart for patients in the whole cohort and across disease aetiologies. The 8-variable KFRE has previously been shown to have a slightly better risk prediction compared to the 4-variable KFRE [4] and we hypothesised that the 8-variable KFRE may have a better performance given its extended parameters captures abnormalities more prevalent in advanced CKD. However, our finding suggests the 4-variable KFRE is more than adequate for risk prediction in this patient group, likely due to the important predictive power of eGFR and albuminuria at later stages of CKD. In this regard, by using less variables, the 4-variable KFRE presents an attractively accessible tool for estimating future risk of ESRD across CKD stages 3a-5.

### The KFRE has good discrimination for 2- and 5-year risk prediction

We show that the 4-variable KFRE had good discrimination in the whole cohort for prediction of ESRD at 2- and 5-years with AUCs of 0.796 (95% CI 0.762–0.831) and 0.773 (95% CI 0.736–0.810) respectively. These were slightly lower than the AUC of 0.83 (95% CI 0.81–0.85) at 2-years and 0.81 (95% CI 0.77–0.84) at 5-years in the report by Hundemer et al. [7] but the studies share similarly excellent discrimination for certain disease aetiologies such as diabetic nephropathy, hypertensive nephropathy and glomerulonephritis at the 2-year time-point.

### Calibration showed an overestimation of risk at 5-years in the whole cohort but there was consistent underestimation of risk in patients with ADPKD at 2- and 5-years

With respect to calibration, we found the KFREs underestimated risk at 2-years and overestimated risk at 5-years, especially in patients with higher predicted risk scores. The overestimation of risk at 5-years is likely explained by the death-censored analysis, which was undertaken as per the original KFRE development study [4]. We show in our sensitivity analysis that this approach does lead to an overestimation of the observed events of ESRD over time as compared to an analysis that treats death as a competing event, which has been shown to be the case in a recent analysis [16].

In contrast to the findings by Hundemer et al. [7], we highlight that the KFRE had a poorer performance in patients with ADPKD in our cohort. For instance, ADPKD demonstrated the lowest AUC values amongst the disease categories across the 4- and 8-variable KFREs. Interestingly, this underperformance was statistically significant when compared with the discriminative ability of the 8-variable KFRE in patients with diabetic nephropathy and glomerulonephritis within the 2-year analysis. This latter finding provides further compelling weight towards reliance on the 4-variable KFRE, especially in those with ADPKD. However, with respect to calibration, all the KFREs consistently underestimated the risk of ESRD in this patient group, which is of particular relevance given that patients with ADPKD had the highest proportion of ESRD at 2- and 5-years (Table 2). Renal progression in ADPKD is notably different to other disease aetiologies in that it can be characterised by rapid rates of decline, often in a linear fashion [17], and this reflects the genetically pre-determined expansion of renal cysts that destroy healthy parenchyma over time, and which is not influenced by modification of risk factors such as uACR. This disease mechanism is evidently not well predicted through the variables within the KFRE alone. Interestingly, there is emerging evidence that suggests that total kidney volume, calculated on ultrasonographic parameters, can be combined with the KFRE to afford better risk prediction performance in ADPKD [18] but further work will be required to corroborate these findings. For now, based on our findings, we would argue using the KFREs with caution in patients with ADPKD.

### Overall, the KFREs demonstrate better clinical utility than relying on eGFR to guide further management

Our validation study offers novel insight into the clinical impact of the 4- and 8-variable KFREs in an advanced CKD population by assessing clinical utility through decision curve analyses, which incorporates the measures of discrimination and calibration [14]. We show that intervening on patients on the basis of a KFRE assessment was the optimal model of choice compared to using eGFR cut-offs of < 20 ml/min/1.73m^2^ and < 15 ml/min/1.73m^2^ over a range of appropriate threshold probabilities. Specifically, the 2-year KFREs were superior at the 40% ESRD threshold and the 5-year KFREs were superior at the 50% ESRD threshold, both thresholds identified in the literature as being relevant to guiding further care [5, 6]. This provides evidence for the overall accuracy of the KFREs in advanced CKD and suggests they can be relied upon more than eGFR alone to support clinical decisions.

#### Clinical implications and future perspectives

We consider that there are two important roles in the application of the KFREs in multidisciplinary advanced care clinics: risk communication and planning for RRT. Communicating risk to patients is important as it provides an avenue to engage, counsel and potentially modify behaviour for patients at high-risk. Using the KFRE has been shown to be far more accurate than subjectively determining patients’ risk: in a prospective study of 257 patients with CKD stages 3–5, the KFRE better matched 2-year outcomes of ESRD than the predicted estimates from nephrologists and patients, who both tended to overestimate risk [19].

With accurate risk prediction comes the corollary of using thresholds to plan for RRT in a timely manner. Our AKCS clinic prioritises pre-emptive transplantation given that this affords the best long-term outcomes [20]. Recognising that it is important to factor in time for medical optimisation and thereafter the time waiting for a transplant, especially from a deceased donor, the risk threshold for referral for transplant work-up becomes automatically lower. Arguably, a ‘treatment-for-all’ strategy (ie. immediate referral for transplant work-up in a suitable patient) is best for patients upon arrival in the AKCS clinic. However, there is potential to refine the approach to planning for arteriovenous (AV) fistula formation, which guidelines recommend should be undertaken around 6 months prior to dialysis initiation [21]. Our work highlights that the KFRE could be employed in those with ≥40% ESRD risk over 2-years to help prioritise patients appropriately, especially given the median time-to-ESRD was 11 months (6-19 months) in this subset of patients. This could help reduce the uncertainty of the optimal time to refer patients for AV fistula formation, whist reducing the morbidity associated with AV fistula creation in patients for whom it is not yet needed [22].

In addition, appropriately timing the initial referral and triage into the AKCS clinic would also be valuable to maximise this treatment opportunity. Indeed, a proposed KFRE cut-off of ≥10% at 2-years has been reported to select patients into multidisciplinary advanced care clinics, a strategy that has captured high-risk patients with an eGFR> 30 ml/min/1.73m^2^. This approach has provided significant cost-savings through the reallocation of resources to those most likely to progress to ESRD [6] and was valued to be of benefit from a qualitative analysis of clinicians and patients’ perspectives [23]. Further prospective work with quality improvement initiatives or cluster randomised trials would be helpful to gauge how successful the KFRE is at achieving higher rates of pre-emptive transplantation or mature AV fistula formation in those who progress to ESRD.

A limiting step in the routine use of the KFRE at our institution is the need for conversion of uPCR to uACR and a change in practice would ideally be needed to help integrate an immediate and accessible risk score into our electronic patient record. It is also important to acknowledge that risk scores obtained by the KFRE should only be used along with clinical judgement given the complexities of care in advanced CKD, where shared decision-making regarding future RRT needs to take account of patients’ preferences, their comorbidities, symptoms and the competing risk of death prior to ESRD.

#### Strengths and limitations

Our study provides for the first time a comprehensive, independent, geographical validation of both the 4- and 8-variable KFREs in advanced CKD in a UK-based cohort with specific evaluation of discrimination, calibration and clinical utility. We also provide insight into the applicability of the KFRE in an advanced kidney care clinic setting by focussing attention on the importance of communicating risk to patients, facilitating pre-emptive transplant and planning for AV fistula formation. This will be of significance to institutions who are considering the merits of using the KFRE in their practices.

There are important limitations to our work. Firstly, there may have been misclassification of patients with diabetic or hypertensive nephropathy as the majority of these patients had not undergone a renal biopsy. This, however, is reflective of routine practice where the clinical probability of these particular diseases typically outweighs the risk of undergoing a biopsy for diagnostic confirmation. Nonetheless, our patient characteristics are in keeping with what we would expect in specific disease aetiologies, notably with higher levels of albuminuria in diabetic nephropathy compared with hypertensive nephropathy. Secondly, we were dependent on converting uPCR to uACR for all our patients, which may have impacted the predicted risk scores, but the online conversion tool we used has been shown to be effective with KFRE calculations. Finally, our study cohort originated from a single-centre and was largely Caucasian, which limits the generalisability of our results to other diverse clinical settings.

## Conclusions

The KFRE is an accessible and useful tool for risk prediction in patients with advanced CKD and in different disease aetiologies. Based on its beneficial clinical utility, the KFRE could be used in multidisciplinary advanced kidney care clinics to help deliver personalised and accurate care. The communication of risk scores can help facilitate early discussion to optimise living donor pre-emptive transplant and assist in decisions on the timing for dialysis access formation. Its use is also likely to be beneficial when managing patients at earlier stages of CKD to identify those at risk of rapid progression. Prospective data would be welcome to highlight the effectiveness of the KFRE in these patient groups, which would help herald a paradigm shift towards the routine use of objective risk-based assessments in delivering optimal CKD care.

## Supplementary Information

**Additional file 1.** The 4- and 8-variable Kidney Failure Risk Equation calculations for the 2- and 5-year predicted risk of ESRD and the formula to convert uPCR to uACR.

**Additional file 2.** TRIPOD checklist for reporting of validation studies.

**Additional file 3.** Baseline characteristics of patients within the 5-year KFRE analysis.

**Additional file 4.** Comparison of the SKS study cohort to the KFRE development cohort.

**Additional file 5.** AUC comparison between disease aetiologies for the 4- and 8- variable KFRE.

**Additional file 6.** Tabulated overall calibration for 4- and 8-variable KFRE according to disease aetiology.

**Additional file 7.** Sensitivity analysis to show probability of events with 1-Kaplan Meier estimate (death as a censored event) compared with cumulative incidence function (death as a competing event).

**Additional file 8.** Raw dataset to perform the analysis.

## Data Availability

The raw dataset is provided in Additional file 8.

## Abbreviations

ADPKD: Autosomal dominant polycystic kidney disease
AKCS: Advanced Kidney Care Service
AUC: Area under the curve
AV: Arteriovenous
CI: Confidence interval
CKD: Chronic kidney disease
eGFR: Estimated glomerular filtration rate
ESRD: End-stage renal disease
KFRE: Kidney Failure Risk Equation
ROC: Receiver operator characteristic curve
RRT: Renal replacement therapy
uACR: Urine albumin:creatinine ratio
UK: United Kingdom
uPCR: Urine protein:creatinine ratio
