# Supplementary material for: A validation study of the kidney failure risk equation in advanced chronic kidney disease according to disease aetiology with evaluation of discrimination, calibration and clinical utility
Source: BMC Nephrol. 2021 May 24;22:194. doi: 10.1186/s12882-021-02402-1 (PMC8147075; doi:10.1186/s12882-021-02402-1)
Supplement: Supplementary file 1 — Additional file 1. The 4- and 8-variable Kidney Failure Risk Equation calculations for the 2- and 5-year predicted risk of ESRD and the formula to convert uPCR to uACR. [file 12882_2021_2402_MOESM1_ESM.docx]

**A validation study of the kidney failure risk equation in advanced chronic kidney disease according to disease aetiology with evaluation of discrimination, calibration and clinical utility**

Ibrahim Ali, Rosemary L. Donne, Philip A. Kalra

**The 4- and 8-variable Kidney Failure Risk Equation calculations for the 2- and 5-year predicted risk of ESRD**

4-variable 2-year calibrated non-North American equation:

1 - 0.9832^exp(-0.2201 x (age/10 – 7.036) + 0.2467 x (male – 0.5642) – 0.5567 x (eGFR/5 – 7.222) + 0.4510 x (logACR – 5.137))

8-variable 2-year calibrated non-North American equation

1 - 0.9827 ^ exp(-0.1992 x (age/10 – 7.036) + 0.1602 (male – 0.5642) – 0.4919 x (eGFR/5 – 7.222) + 0.3364 x (logACR – 5.137) – 0.3441 x (albumin – 3.997) + 0.2604 x (phosphate – 3.916) – 0.07354 x (bicarbonate – 25.57) – 0.2228 x (calcium – 9.355))

4-variable 5-year calibrated non-North American equation:

1 - 0.9365^exp(-0.2201 x (age/10 – 7.036) + 0.2467 x (male – 0.5642) – 0.5567 x (eGFR/5 – 7.222) + 0.4510 x (logACR – 5.137))

8-variable 5-year calibrated non-North American equation:

1 - 0.9245 ^ exp(-0.1992 x (age/10 – 7.036) + 0.1602 (male – 0.5642) – 0.4919 x (eGFR/5 – 7.222) + 0.3364 x (logACR – 5.137) – 0.3441 x (albumin – 3.997) + 0.2604 x (phosphate – 3.916) – 0.07354 x (bicarbonate – 25.57) – 0.2228 x (calcium – 9.355))

In the above equations,

Age is the patient’s age in years, at the time of the laboratory measurements.

Male is equal to 1, otherwise 0.

eGFR is the estimated glomerular filtration rate in ml/min/1.73m^2^, calculated using the CKD-EPI equation.

LogACR is the natural logarithm of the urine albumin:creatinine ratio, measured in mg/g.

Albumin is serum albumin measured in mg/dl.

Phosphate is serum phosphate is measured in mg/dl.

Bicarbonate is serum bicarbonate measured in mEq/L

Calcium is serum calcium measured in mg/dl.

**Formula to convert uPCR to uACR**

exp(5.2659+0.2934*LN(MIN(uPCR/50,1))+1.5643*LN(MAX(MIN(uPCR/500,1),0.1)))+1.1109*LN(MAX(uPCR/500,1)))-0.0773*(female)+0.0797*(diabetic)+0.1265*(hypertensive))

In the above equation,

uPCR is measured in mg/g.

Female=1

Diabetic=1

Hypertensive=1
