# Supplementary material for: A validation study of the kidney failure risk equation in advanced chronic kidney disease according to disease aetiology with evaluation of discrimination, calibration and clinical utility
Source: BMC Nephrol. 2021 May 24;22:194. doi: 10.1186/s12882-021-02402-1 (PMC8147075; doi:10.1186/s12882-021-02402-1)
Supplement: Supplementary file 2 — Additional file 2. TRIPOD checklist for reporting of validation studies. [file 12882_2021_2402_MOESM2_ESM.docx]

**A validation study of the kidney failure risk equation in advanced chronic kidney disease according to disease aetiology with evaluation of discrimination, calibration and clinical utility**

Ibrahim Ali, Rosemary L. Donne, Philip A. Kalra

**TRIPOD checklist for reporting of validation studies**

| **Section/topic** | **Item** | **Checklist item** | **Page** |
| --- | --- | --- | --- |
| **Title and abstract** | | | |
| Title | **1** | Identify the study as developing and/or validating a multivariable prediction model, the target population, and the outcome to be predicted. | **1** |
| Abstract | **2** | Provide a summary of objectives, study design, setting, participants, sample size, predictors, outcome, statistical analysis, results, and conclusions. | **2-3** |
| **Introduction** | | | |
| Background and objectives | **3a** | Explain the medical context (including whether diagnostic or prognostic) and rationale for developing or validating the multivariable prediction model, including references to existing models. | **4** |
|  | **3b** | Specify the objectives, including whether the study describes the development or validation of the model or both. | **5** |
| **Methods** | | | |
| Source of data | 4a | Describe the study design or source of data (e.g., randomized trial, cohort, or registry data), separately for the development and validation data sets, if applicable. | **5** |
|  | 4b | Specify the key study dates, including start of accrual; end of accrual; and, if applicable, end of follow-up. | **6-7** |
| Participants | 5a | Specify key elements of the study setting (e.g., primary care, secondary care, general population) including number and location of centres. | **5** |
|  | 5b | Describe eligibility criteria for participants. | **6** |
|  | 5c | Give details of treatments received, if relevant. | **N/A** |
| Outcome | 6a | Clearly define the outcome that is predicted by the prediction model, including how and when assessed. | **7** |
|  | 6b | Report any actions to blind assessment of the outcome to be predicted. | **N/A** |
| Predictors | 7a | Clearly define all predictors used in developing or validating the multivariable prediction model, including how and when they were measured. | **6** |
|  | 7b | Report any actions to blind assessment of predictors for the outcome and other predictors. | **N/A** |
| Sample size | 8 | Explain how the study size was arrived at. | **6** |
| Missing data | 9 | Describe how missing data were handled (e.g., complete-case analysis, single imputation, multiple imputation) with details of any imputation method. | **N/A** |
| Statistical analysis methods | 10c | For validation, describe how the predictions were calculated. | **Additional file 1** |
|  | 10d | Specify all measures used to assess model performance and, if relevant, to compare multiple models. | **7-8** |
|  | 10e | Describe any model updating (e.g., recalibration) arising from the validation, if done. | **N/A** |
| Risk groups | 11 | Provide details on how risk groups were created, if done. | **N/A** |
| Development vs. validation | 12 | For validation, identify any differences from the development data in setting, eligibility criteria, outcome, and predictors. | **5** |
| **Results** | | | |
| Participants | 13a | Describe the flow of participants through the study, including the number of participants with and without the outcome and, if applicable, a summary of the follow-up time. A diagram may be helpful. | **Fig. 1** |
|  | 13b | Describe the characteristics of the participants (basic demographics, clinical features, available predictors), including the number of participants with missing data for predictors and outcome. | **Table 1** |
|  | 13c | For validation, show a comparison with the development data of the distribution of important variables (demographics, predictors and outcome). | **Additional file 4** |
| Model performance | 16 | Report performance measures (with CIs) for the prediction model. | **Tables 3-4; Fig. 3** |
| Model-updating | 17 | If done, report the results from any model updating (i.e., model specification, model performance). | **N/A** |
| **Discussion** | | | |
| Limitations | 18 | Discuss any limitations of the study (such as nonrepresentative sample, few events per predictor, missing data). | **20** |
| Interpretation | 19a | For validation, discuss the results with reference to performance in the development data, and any other validation data. | **15-18** |
|  | 19b | Give an overall interpretation of the results, considering objectives, limitations, results from similar studies, and other relevant evidence. | **15-18** |
| Implications | 20 | Discuss the potential clinical use of the model and implications for future research. | **18-19** |
| **Other information** | | | |
| Supplementary information | 21 | Provide information about the availability of supplementary resources, such as study protocol, Web calculator, and data sets. | **Additional files** |
| Funding | 22 | Give the source of funding and the role of the funders for the present study. | **N/A** |
