# Supplementary material for: A validation study of the kidney failure risk equation in advanced chronic kidney disease according to disease aetiology with evaluation of discrimination, calibration and clinical utility
Source: BMC Nephrol. 2021 May 24;22:194. doi: 10.1186/s12882-021-02402-1 (PMC8147075; doi:10.1186/s12882-021-02402-1)
Supplement: Supplementary file 3 — Additional file 3. Baseline characteristics of patients within the 5-year KFRE analysis. [file 12882_2021_2402_MOESM3_ESM.docx]

**A validation study of the kidney failure risk equation in advanced chronic kidney disease according to disease aetiology with evaluation of discrimination, calibration and clinical utility**

Ibrahim Ali, Rosemary L. Donne, Philip A. Kalra

**Baseline characteristics of patients within the 5-year KFRE analysis**

| **Variable** | **Whole cohort** | **Diabetic nephropathy** | **Hypertensive nephropathy** | **GN** | **ADPKD** | **Other diseases** |
| --- | --- | --- | --- | --- | --- | --- |
| Patient numbers | 613 | 140 | 115 | 75 | 49 | 234 |
| Age, years | 68.7 (56.6-77.4) | 66.2 (56.4-74.7) | 76.3 (69.3-81.4) | 59.4 (45.3-72.0) | 54.2 (46.4-64.4) | 71.3 (61.7-79.2) |
| Male, *n* (%) | 386 (63) | 95 (68) | 74 (64) | 49 (65) | 30 (61) | 138 (59) |
| Caucasian, *n* (%) | 578 (94) | 130 (93) | 111 (97) | 69 (92) | 49 (80) | 220 (94) |
| Hypertension, *n* (%) | 595 (97) | 138 (99) | 115 (100) | 75 (100) | 46 (94) | 221 (94) |
| Diabetes mellitus, *n* (%) | 246 (40) | 140 (100) | 32 (28) | 15 (20) | 2 (4) | 57 (24) |
|  |  |  |  |  |  |  |
| **Laboratory values** |  |  |  |  |  |  |
| ^1^eGFR, ml/min/1.73m^2^ | 15 (12-18) | 16 (13-19) | 15 (12-18) | 16 (12-19) | 16 (12-18) | 15 (13-18) |
| ^†^urine albumin:creatinine ratio, mg/g | 386 (81-1263) | 734 (144-2220) | 166 (42-596) | 1166 (424-2517) | 143 (47-328) | 357 (81-914) |
| ^‡^Calcium, mg/dL | 9.32 (8.92-9.72) | 9.40 (9.00-9.76) | 9.24 (8.76-9.68) | 9.28 (8.96-9.76) | 9.32 (8.88-9.56) | 9.34 (8.96-9.72) |
| ^‡^Phosphate, mg/dL | 3.91 (3.35-4.50) | 4.00 (3.43-4.62) | 3.78 (3.29-4.34) | 4.22 (3.46-4.84) | 4.03 (3.57-4.34) | 3.81 (3.29-4.37) |
| Bicarbonate, mEq/L | 21.3 (19.3-23.7) | 22.0 (20.2-24.4) | 21.5 (19.7-23.6) | 20.7 (19.0-22.9) | 20.7 (18.8-23.5) | 21.3 (19.1-23.7) |
| ^¶^Albumin, g/dL | 4.2 (3.9-4.4) | 4.0 (3.7-4.2) | 4.2 (4.0-4.4) | 4.0 (3.7-4.4) | 4.4 (4.2-4.6) | 4.2 (4.0-4.4) |
|  |  |  |  |  |  |  |
| **KFRE scores** |  |  |  |  |  |  |
| 4-variable 2-year score, % | 24 (11-42) | 31 (13-52) | 15 (7-30) | 38 (22-65) | 20 (12-36) | 22 (9-37) |
| 4-variable 5-year score, % | 65 (36-88) | 76 (41-94) | 46 (24-74) | 85 (62-98) | 59 (39-83) | 62 (33-83) |
| 8-variable 2-year score, % | 21 (10-40) | 23 (12-50) | 13 (7-28) | 30 (18-66) | 22 (13-31) | 19 (10-34) |
| 8-variable 5-year score, % | 65 (38-90) | 69 (45-96) | 47 (27-77) | 79 (59-99) | 66 (46-81) | 62 (37-84) |

Continuous data expressed as median (interquartile range) and categorical data as number (percentage). ^*^eGFR was calculated using the Chronic Kidney Disease Epidemiology Collaboration (CKD-EPI) equation. ^†^urine albumin:creatinine ratios were acquired by converting urine protein:creatinine ratios using an online calculator [9] and thereafter switching units from mg/mmol to mg/g by multiplying values by 8.84. ^‡^Calcium and phosphate were measured in mmol/L and converted to mg/dL by multiplying values by 4 and 3.1 respectively. ^¶^Albumin was measured in g/L and converted to g/dL by dividing by 10.

**Abbreviations**: GN (glomerulonephritis); ADPKD (autosomal dominant polycystic kidney disease); eGFR (estimated glomerular filtration rate); ESRD (end-stage renal disease); KFRE (Kidney Failure Risk Equation)
