# Supplementary material for: A validation study of the kidney failure risk equation in advanced chronic kidney disease according to disease aetiology with evaluation of discrimination, calibration and clinical utility
Source: BMC Nephrol. 2021 May 24;22:194. doi: 10.1186/s12882-021-02402-1 (PMC8147075; doi:10.1186/s12882-021-02402-1)
Supplement: Supplementary file 4 — Additional file 4. Comparison of the SKS study cohort to the KFRE development cohort. [file 12882_2021_2402_MOESM4_ESM.docx]

**A validation study of the kidney failure risk equation in advanced chronic kidney disease according to disease aetiology with evaluation of discrimination, calibration and clinical utility**

Ibrahim Ali, Rosemary L. Donne, Philip A. Kalra

**Comparison of the SKS study cohort to the KFRE development cohort**

|  | **2-year validation cohort in SKS**  **(n = 743)** | **Original KFRE development cohort**  **(n = 3449)** |
| --- | --- | --- |
| **Age, years** | 66 (15) | 70 (14) |
| **Male, *n* (%)** | 462 (62) | 1946 (56) |
| **eGFR, ml/min/1.73m^2^** | 16 (4) | 36 (13) |
| **Serum bicarbonate, mEq/L** | 22 (4) | 26 (4) |
| **Serum calcium, mg/dL** | 9.3 (0.6) | 9.4 (0.6) |
| **Serum phosphate, mg/dL** | 4.0 (1.0) | 4.0 (0.9) |
| **Serum albumin, mg/dL** | 4.1 (0.4) | 4.0 (0.5) |
| **Urine albumin:creatinine ratio, mg/g** | 409 (1271) | 93 (378) |
| **2-year events of ESRD, *n* (%)** | 257 (35) | 386 (11) |

All continuous variables are presented as means (standard deviation) except for urine albumin:creatinine ratio, which is shown as median (interquartile range).

**Abbreviations**: KFRE (Kidney Failure Risk Equation); eGFR (estimated glomerular filtration rate); SKS (Salford Kidney Study)
