# Supplementary material for: A validation study of the kidney failure risk equation in advanced chronic kidney disease according to disease aetiology with evaluation of discrimination, calibration and clinical utility
Source: BMC Nephrol. 2021 May 24;22:194. doi: 10.1186/s12882-021-02402-1 (PMC8147075; doi:10.1186/s12882-021-02402-1)
Supplement: Supplementary file 5 — Additional file 5. AUC comparison between disease aetiologies for the 4- and 8- variable KFRE. [file 12882_2021_2402_MOESM5_ESM.docx]

**A validation study of the kidney failure risk equation in advanced chronic kidney disease according to disease aetiology with evaluation of discrimination, calibration and clinical utility**

Ibrahim Ali, Rosemary L. Donne, Philip A. Kalra

**AUC comparison between disease aetiologies for the 4-variable KFRE**

|  | **4-variable 2-year risk** | | **4-variable 5-year risk** | |
| --- | --- | --- | --- | --- |
|  | **Difference in AUC** | **p-value** | **Difference in AUC** | **p-value** |
| **Diabetes vs. HTN** | 0.009 | 0.88 | 0.009 | 0.89 |
| **Diabetes vs. GN** | 0.008 | 0.88 | 0.028 | 0.70 |
| **Diabetes vs. ADPKD** | 0.137 | 0.06 | 0.183 | 0.21 |
| **Diabetes vs. Other** | 0.073 | 0.10 | 0.007 | 0.88 |
| **HTN vs. GN** | 0.001 | 0.99 | 0.019 | 0.80 |
| **HTN vs. ADPKD** | 0.128 | 0.12 | 0.174 | 0.24 |
| **HTN vs. Other** | 0.064 | 0.27 | 0.016 | 0.77 |
| **GN vs. ADPKD** | 0.129 | 0.10 | 0.155 | 0.31 |
| **GN vs. Other** | 0.065 | 0.22 | 0.035 | 0.59 |
| **ADPKD vs. Other** | 0.064 | 0.38 | 0.190 | 0.19 |

**AUC comparison between disease aetiologies for the 8-variable KFRE**

|  | **8-variable 2-year risk** | | **8-variable 5-year risk** | |
| --- | --- | --- | --- | --- |
|  | **Difference in AUC** | **p-value** | **Difference in AUC** | **p-value** |
| **Diabetes vs. HTN** | 0.042 | 0.50 | 0.007 | 0.91 |
| **Diabetes vs. GN** | 0.013 | 0.82 | 0.012 | 0.87 |
| **Diabetes vs. ADPKD** | 0.188 | 0.02 | 0.171 | 0.34 |
| **Diabetes vs. Other** | 0.079 | 0.05 | 0.013 | 0.80 |
| **HTN vs. GN** | 0.029 | 0.68 | 0.005 | 0.95 |
| **HTN vs. ADPKD** | 0.146 | 0.10 | 0.164 | 0.36 |
| **HTN vs. Other** | 0.044 | 0.48 | 0.006 | 0.92 |
| **GN vs. ADPKD** | 0.175 | 0.04 | 0.159 | 0.38 |
| **GN vs. Other** | 0.073 | 0.18 | 0.001 | 0.99 |
| **ADPKD vs. Other** | 0.102 | 0.20 | 0.158 | 0.37 |

Pairwise comparisons of the AUCs were undertaken using DeLong’s method [12].

**Abbreviations**: HTN (hypertensive nephropathy); GN (glomerulonephritis); ADPKD (autosomal dominant polycystic kidney disease); AUC (area under receiver operator characteristic curve).
