# Supplementary material for: A validation study of the kidney failure risk equation in advanced chronic kidney disease according to disease aetiology with evaluation of discrimination, calibration and clinical utility
Source: BMC Nephrol. 2021 May 24;22:194. doi: 10.1186/s12882-021-02402-1 (PMC8147075; doi:10.1186/s12882-021-02402-1)
Supplement: Supplementary file 6 — Additional file 6. Tabulated overall calibration for 4- and 8-variable KFRE according to disease aetiology. [file 12882_2021_2402_MOESM6_ESM.docx]

**A validation study of the kidney failure risk equation in advanced chronic kidney disease according to disease aetiology with evaluation of discrimination, calibration and clinical utility**

Ibrahim Ali, Rosemary L. Donne, Philip A. Kalra

**Tabulated overall calibration for 4- and 8-variable KFRE according to disease aetiology**

| **4-variable 2-year KFRE risk prediction** | | |
| --- | --- | --- |
| **Disease** | **Average predicted score, %** | **2-year observed events, %** |
| Whole cohort | 29 | 35 |
| Diabetic nephropathy | 35 | 37 |
| Hypertensive nephropathy | 21 | 26 |
| Glomerulonephritis | 43 | 47 |
| ADPKD | 21 | 63 |
| Other diseases | 26 | 28 |

| **8-variable 2-year KFRE risk prediction** | | |
| --- | --- | --- |
| **Disease** | **Average predicted score, %** | **2-year observed events, %** |
| Whole cohort | 24 | 35 |
| Diabetic nephropathy | 32 | 37 |
| Hypertensive nephropathy | 22 | 26 |
| Glomerulonephritis | 44 | 47 |
| ADPKD | 22 | 63 |
| Other diseases | 25 | 28 |

| **4-variable 5-year KFRE risk prediction** | | |
| --- | --- | --- |
| **Disease** | **Average predicted score, %** | **5-year observed events, %** |
| Whole cohort | 61 | 54 |
| Diabetic nephropathy | 67 | 56 |
| Hypertensive nephropathy | 49 | 39 |
| Glomerulonephritis | 77 | 68 |
| ADPKD | 58 | 90 |
| Other diseases | 59 | 48 |

| **8-variable 5-year KFRE risk prediction** | | |
| --- | --- | --- |
| **Disease** | **Average predicted score, %** | **5-year observed events, %** |
| Whole cohort | 62 | 54 |
| Diabetic nephropathy | 67 | 56 |
| Hypertensive nephropathy | 52 | 39 |
| Glomerulonephritis | 77 | 68 |
| ADPKD | 62 | 90 |
| Other diseases | 60 | 48 |

**Abbreviations**: ADPKD (autosomal dominant polycystic kidney disease)
