# Supplementary material for: A validation study of the kidney failure risk equation in advanced chronic kidney disease according to disease aetiology with evaluation of discrimination, calibration and clinical utility
Source: BMC Nephrol. 2021 May 24;22:194. doi: 10.1186/s12882-021-02402-1 (PMC8147075; doi:10.1186/s12882-021-02402-1)
Supplement: Supplementary file 7 — Additional file 7. Sensitivity analysis to show probability of events with 1-Kaplan Meier estimate (death as a censored event) compared with cumulative incidence function (death as a competing event). [file 12882_2021_2402_MOESM7_ESM.docx]

**A validation study of the kidney failure risk equation in advanced chronic kidney disease according to disease aetiology with evaluation of discrimination, calibration and clinical utility**

Ibrahim Ali, Rosemary L. Donne, Philip A. Kalra

**Sensitivity analysis to show probability of events with 1-Kaplan Meier estimate (death as a censored event) compared with cumulative incidence function (death as a competing event)**

**4-variable 2-year KFRE**


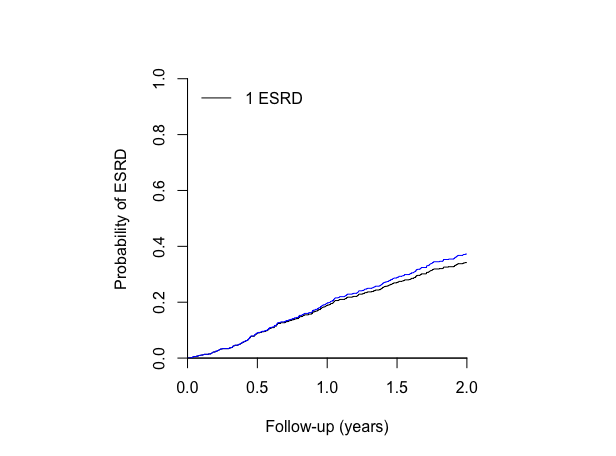


1-Kaplan Meier estimate

Cumulative incidence function

Number at risk

Cumulative events

743 535 360

0 150 257


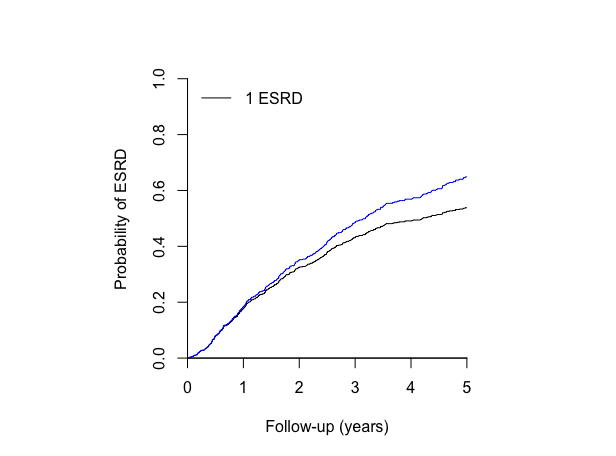
**4-variable 5-year KFRE**

613 455 335 232 166 111

0 117 200 267 303 331

Number at risk

Cumulative events

1-Kaplan Meier estimate

Cumulative incidence function
